# Crystal structure of bromido(η^6^-1-isopropyl-4-methylbenzene)(7-oxocyclohepta-1,3,5-trien-1-olato-κ^2^ *O*,*O*′)osmium

**DOI:** 10.1107/S2056989018001391

**Published:** 2018-02-02

**Authors:** Hadley S. Clayton, Kgaugelo C. Tapala, Andreas Lemmerer

**Affiliations:** a Chemistry Department, University of South Africa, Pretoria, 0003, South Africa; b Molecular Sciences Institute, School of Chemistry, University of the Witwatersrand, Private Bag, PO WITS, 2050, Johannesburg, South Africa

**Keywords:** crystal structure, osmium(II) complex, tropolonato, cymene ligand

## Abstract

In the title compound, [OsBr(C_10_H_14_)(C_7_H_5_O_2_)], the central Os^II^ ion is ligated by a hexa­haptic η^6^ *p*-cymene ring, a Br^−^ ligand and two O atoms of a chelating tropolonate group. The *p*-cymene ligand presents more than one conformation, giving rise to a discrete disorder, which was modelled with two different orientations with occupancy values of 0.561 (15) and 0.439 (15). The crystal packing features C—H⋯O and C—H⋯Br hydrogen bonding. Aromatic π–π stacking inter­actions are also observed between adjacent non-benzenoid aromatic tropolone rings.

## Abstract 2

In the title compound, the central Os^II^ ion is ligated by a hexa­haptic η^6^ *p*-cymene ring, a Br^−^ ligand and two O atoms of a chelating tropolonate group. The *p*-cymene ligand presents more than one conformation, giving rise to a discrete disorder, which was modelled with two different orientations with occupancy values of 0.561 (15) and 0.439 (15).

## Chemical context   

The chemistry of half-sandwich organometallic Os^II^–arene complexes with O-donor ligands has drawn considerable inter­est because of their potential application as anti­cancer agents (Zhang & Sadler, 2017 ▸). In particular, several complexes of this type with *O*,*O*- and *N*,*O*-chelating ligands have been investigated (Hanif *et al.*, 2010 ▸; van Rijt *et al.*, 2009 ▸). While the complexes with *N*,*O*-ligands have shown *in vitro* anti­cancer activity comparable to *Cisplatin*, the benchmark anti­cancer metallopharmaceutical, complexes with *O*,*O*-ligands exhibit low activity. This has been attributed to the poor stability of these complexes in aqueous solution and the formation of inactive hy­droxy-bridged dimers (Hanif *et al.*, 2014 ▸). The mechanism of the cytotoxic action of the Os^II^–arene complexes is generally thought to involve hydrolysis of the Os—*X* bond (where *X* = a halide ligand) to generate an active Os–OH_2_ species, which binds to biomolecules leading to cellular dysfunction and consequently triggering apoptosis. While the anti­cancer activity of the Os^II^–arene complexes has often been compared to that of their Ru analogues, no defin­itive structure–activity relationship has yet been elucidated. In addition, the Os^II^–arene complexes appear to have an altered pharmacological profile in comparison with the ruthenium complexes (Bruijnincx & Sadler, 2009 ▸). As part of our studies in this area, single-crystal X-ray diffraction was used to determine the structure of the title compound, (I).

## Structural commentary   

**Figure 1:**
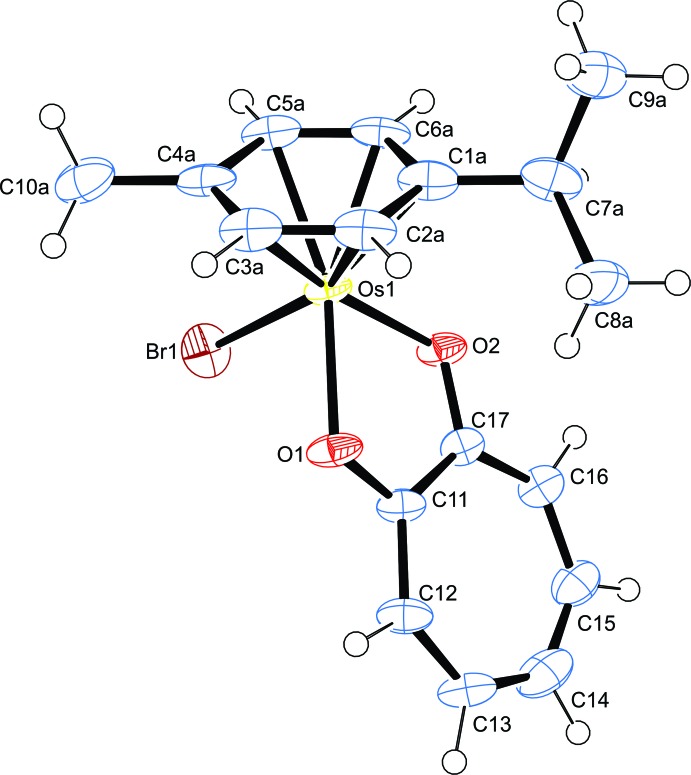
The mol­ecular structure of (I) showing 50% displacement ellipsoids. Only one orientation of the disordered benzene ring is shown.

The mol­ecular structure of (I) is shown in Fig. 1 ▸ and selected geometrical data are presented in Table 1 ▸. The complex adopts a ‘three-legged piano-stool’ geometry, where the η^6^-coord­inated arene ring is present as the seat, and the two O atoms of the tropolonate ligand along with the bromido ligand as the three legs of the stool.
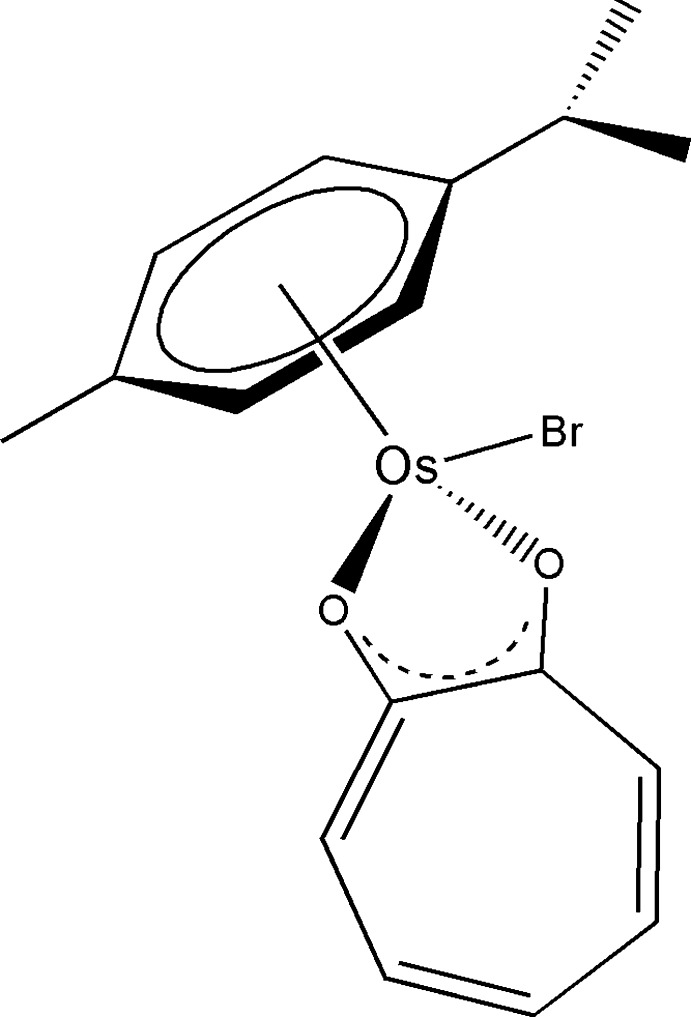


The tropolonato anion is chelated to the metal centre, forming a five-membered OsO_2_C_2_ ring, which is almost planar, with the tight bite angle [76.3 (2)°] of the tropolonate chelate resulting in a distorted pseudo-octa­hedral coordination sphere. The rigid tropolonate ligand backbone is made up of an almost planar seven-membered ring consisting of conjugated sp^2^ carbon atoms. The Os—O bond lengths [2.071 (6) and 2.088 (6) Å] are similar to those of the related ruthenium compound (*ca* 2.1 Å) published previously (Dwivedi *et al.*, 2016 ▸). The isobidentate nature of the OsO_2_C_2_ moiety is evidence of delocalization of the C=O bonds of the tropolone ligand upon coordination [C11—O1 = 1.303 (11), C17—O2 = 1.299 (11)Å]. The aromatic ring of the *p*-cymene ligand appears almost planar, with the displacement of the arene ring centroid from the Os^II^ center [1.676 Å] being comparable with other similar complexes (Peacock *et al.*, 2007 ▸; Kandioller *et al.* 2013 ▸).

## Supra­molecular features   

**Figure 2:**
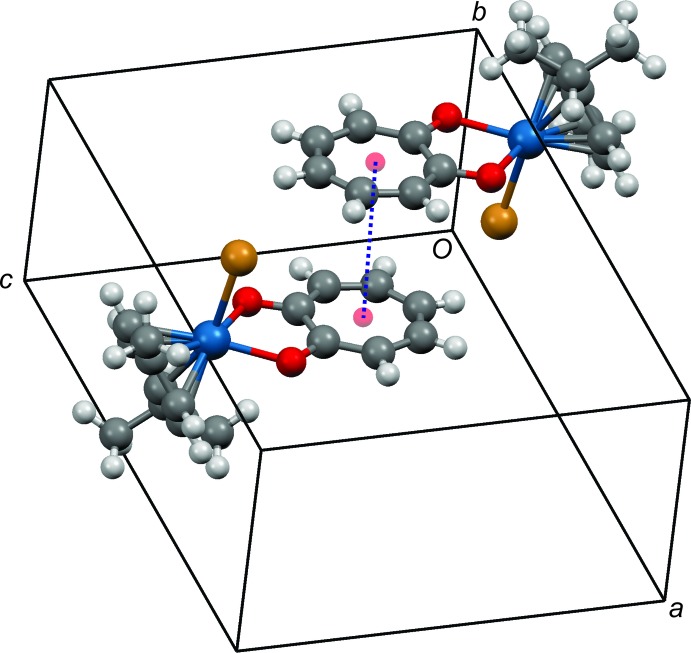
Detail of the packing of (I) showing aromatic π–π stacking between the seven-membered rings as a blue dashed line.

In the crystal, the coordinated O atoms of the tropolonate ligand accept weak C—H⋯O inter­actions (Table 2 ▸) from the *p*-cymene ring in the range 2.40–2.72 Å, which contribute to the crystal packing. In addition, the bromide ion acts as a hydrogen-bond acceptor, forming C—H⋯Br hydrogen bonds with a C—H group from the arene ring of an adjacent mol­ecule. There is also a π–π stacking inter­action between the tropolone ligands with the plane-to-plane distances of the stacked aromatic ring moieties at 3.895 Å (Fig. 2 ▸).

## Database survey   

A search of the Cambridge Structural Database (Version 5.38, update February 2017; Groom *et al.*, 2016 ▸) for related structures revealed that the isostructural ruthenium complex, [(η^6^-*p*-cymene)Ru(trop)Cl] (OTIMOV; Dwivedi *et al.*, 2016 ▸), and similar osmium complexes (QEYXIC; Peacock *et al.*, 2007 ▸ and BENYUQ; Kandioller *et al.*, 2013 ▸) have been reported.

## Synthesis and crystallization   

All synthetic procedures were carried out using standard Schlenk techniques under an atmosphere of argon. The osmium dimer [Os(η^6^-*p*-cymene)Br_2_]_2_ (1.037 g, 1.07 mmol) and sodium tropolonate (0.448 g, 3.11 mmol) were suspended in methanol (100 ml). The suspension was stirred at room temperature overnight to give a dark-brown solution. The solution was filtered and the solvent was removed *in vacuo*. The residue was extracted with CH_2_Cl_2_ (80 ml). The solvent was removed under reduced pressure to give the title compound as a red–brown solid. Yield 72% (0.807 g, 1.54 mmol). Red blocks of (I) were obtained by slow evaporation from a concentrated di­chloro­methane solution at room temperature over several days.

## Refinement   

Crystal data, data collection and structure refinement details are summarized in Table 3 ▸. The C1–C10 atoms of the *p*-cymene ligand were modelled as disordered over two orientations with occupancies of 0.561 (15) and 0.439 (15).

## Supplementary Material

Crystal structure: contains datablock(s) global, I. DOI: 10.1107/S2056989018001391/hb7721sup1.cif

Structure factors: contains datablock(s) I. DOI: 10.1107/S2056989018001391/hb7721Isup2.hkl

CCDC reference: 1818437

Additional supporting information:  crystallographic information; 3D view; checkCIF report

## Figures and Tables

**Table 1:** Selected bond lengths (Å)

Os1—C1*A*	2.090 (12)	Os1—C3*B*	2.167 (19)
Os1—C2*A*	2.125 (14)	Os1—C4*B*	2.192 (16)
Os1—C3*A*	2.158 (14)	Os1—C5*B*	2.21 (2)
Os1—C4*A*	2.157 (12)	Os1—C6*B*	2.21 (2)
Os1—C5*A*	2.123 (18)	Os1—O1	2.088 (6)
Os1—C6*A*	2.089 (18)	Os1—O2	2.071 (6)
Os1—C1*B*	2.187 (13)	Os1—Br1	2.5472 (12)
Os1—C2*B*	2.164 (17)		

**Table 2:** Hydrogen-bond geometry (Å, °)

*D*—H⋯*A*	*D*—H	H⋯*A*	*D*⋯*A*	*D*—H⋯*A*
C2*A*—H2*A*⋯O1^i^	0.95	2.40	3.24 (2)	148
C3*B*—H3*B*⋯O1^i^	0.95	2.75	3.38 (2)	124
C5*B*—H5*B*⋯O2^ii^	0.95	2.50	3.25 (2)	136
C6*A*—H6*A*⋯O2^ii^	0.95	2.71	3.39 (2)	124
C5*A*—H5*A*⋯O2^ii^	0.95	2.77	3.39 (2)	124

Symmetry codes: (i) 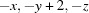; (ii) 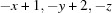.

**Table 3:** Experimental details

Crystal data	
Chemical formula	C_17_H_19_BrO_2_Os
*M*_r_	525.43
Crystal system, space group	Monoclinic, *P*2_1_/*c*
Temperature (K)	173
*a*, *b*, *c* (Å)	11.1574 (5), 14.6104 (7), 10.8342 (5)
β (°)	110.454 (2)
*V* (Å^3^)	1654.78 (13)
*Z*	4
Radiation type	Mo *K*α
μ (mm^−1^)	10.12
Crystal size (mm)	0.12 × 0.10 × 0.05

Data collection	
Diffractometer	Bruker D8 Venture Photon CCD area detector
Absorption correction	Integration (*XPREP*; Bruker, 2016 ▸)
*T*_min_, *T*_max_	0.538, 0.714
No. of measured, independent and observed [*I* > 2σ(*I*)] reflections	58616, 3990, 3614
*R*_int_	0.079
(sin θ/λ)_max_ (Å^−1^)	0.660

Refinement	
*R*[*F* ^2^ > 2σ(*F* ^2^)], *wR*(*F* ^2^), *S*	0.053, 0.118, 1.15
No. of reflections	3990
No. of parameters	259
No. of restraints	384
H-atom treatment	H-atom parameters constrained
Δρ_max_, Δρ_min_ (e Å^−3^)	5.63, −2.07

Computer programs: *APEX3*, *SAINT-Plus* and *XPREP* (Bruker, 2016 ▸), *SHELXS97* (Sheldrick, 2008 ▸) and *SHELXL2014/7* (Sheldrick, 2015 ▸), *ORTEP* for Windows and *WinGX* publication routines (Farrugia, 2012 ▸).

## supplementary crystallographic information

### Crystal data

**Table:**

C_17_H_19_BrO_2_Os	*F*(000) = 992
*M**_r_* = 525.43	*D*_x_ = 2.109 Mg m^−^^3^
Monoclinic, *P*2_1_/*c*	Mo *K*α radiation, λ = 0.71073 Å
Hall symbol: -P 2ybc	Cell parameters from 9941 reflections
*a* = 11.1574 (5) Å	θ = 3.3–28.3°
*b* = 14.6104 (7) Å	µ = 10.12 mm^−^^1^
*c* = 10.8342 (5) Å	*T* = 173 K
β = 110.454 (2)°	Block, red
*V* = 1654.78 (13) Å^3^	0.12 × 0.10 × 0.05 mm
*Z* = 4	

### Data collection

**Table:**

Bruker D8 Venture Photon CCD area detector diffractometer	3614 reflections with *I* > 2σ(*I*)
Graphite monochromator	*R*_int_ = 0.079
ω scans	θ_max_ = 28.0°, θ_min_ = 3.4°
Absorption correction: integration Bruker XPREP (Bruker, 2016)	*h* = −14→14
*T*_min_ = 0.538, *T*_max_ = 0.714	*k* = −19→19
58616 measured reflections	*l* = −14→14
3990 independent reflections	

### Refinement

**Table:**

Refinement on *F*^2^	0 constraints
Least-squares matrix: full	Hydrogen site location: inferred from neighbouring sites
*R*[*F*^2^ > 2σ(*F*^2^)] = 0.053	H-atom parameters constrained
*wR*(*F*^2^) = 0.118	*w* = 1/[σ^2^(*F*_o_^2^) + (0.0137*P*)^2^ + 44.2634*P*] where *P* = (*F*_o_^2^ + 2*F*_c_^2^)/3
*S* = 1.15	(Δ/σ)_max_ < 0.001
3990 reflections	Δρ_max_ = 5.63 e Å^−^^3^
259 parameters	Δρ_min_ = −2.07 e Å^−^^3^
384 restraints	

### Special details

**Table:**

Experimental. Numerical integration absorption corrections based on indexed crystal faces were applied using the XPREP routine (Bruker, 2016)
Geometry. All esds (except the esd in the dihedral angle between two l.s. planes) are estimated using the full covariance matrix. The cell esds are taken into account individually in the estimation of esds in distances, angles and torsion angles; correlations between esds in cell parameters are only used when they are defined by crystal symmetry. An approximate (isotropic) treatment of cell esds is used for estimating esds involving l.s. planes.

### Fractional atomic coordinates and isotropic or equivalent isotropic displacement parameters (Å^2^)

**Table:**

	*x*	*y*	*z*	*U*_iso_*/*U*_eq_	Occ. (<1)
C1A	0.2170 (16)	1.0600 (9)	−0.1234 (11)	0.035 (3)	0.561 (15)
C2A	0.1066 (14)	1.0131 (11)	−0.1297 (16)	0.034 (3)	0.561 (15)
H2A	0.0364	1.0455	−0.1202	0.041*	0.561 (15)
C3A	0.0988 (13)	0.9190 (11)	−0.1497 (16)	0.036 (3)	0.561 (15)
H3A	0.0234	0.887	−0.154	0.043*	0.561 (15)
C4A	0.2015 (16)	0.8718 (9)	−0.1635 (12)	0.035 (3)	0.561 (15)
C5A	0.3120 (14)	0.9186 (13)	−0.1572 (19)	0.034 (3)	0.561 (15)
H5A	0.3822	0.8863	−0.1666	0.041*	0.561 (15)
C6A	0.3197 (13)	1.0127 (13)	−0.1372 (19)	0.033 (3)	0.561 (15)
H6A	0.3951	1.0447	−0.1329	0.039*	0.561 (15)
C7A	0.2286 (19)	1.1633 (10)	−0.1010 (16)	0.040 (3)	0.561 (15)
H7A	0.3211	1.1768	−0.0529	0.048*	0.561 (15)
C8A	0.155 (2)	1.1965 (17)	−0.013 (2)	0.050 (5)	0.561 (15)
H8A1	0.1833	1.1617	0.0691	0.076*	0.561 (15)
H8A2	0.1726	1.2617	0.0059	0.076*	0.561 (15)
H8A3	0.0635	1.1873	−0.0592	0.076*	0.561 (15)
C9A	0.190 (2)	1.2146 (16)	−0.2316 (19)	0.048 (5)	0.561 (15)
H9A1	0.1982	1.2806	−0.2148	0.073*	0.561 (15)
H9A2	0.246	1.1962	−0.2797	0.073*	0.561 (15)
H9A3	0.1011	1.1999	−0.2844	0.073*	0.561 (15)
C10A	0.193 (2)	0.7683 (12)	−0.1851 (17)	0.046 (4)	0.561 (15)
H10A	0.144	0.7411	−0.1345	0.068*	0.561 (15)
H10B	0.2789	0.742	−0.1558	0.068*	0.561 (15)
H10C	0.1493	0.7552	−0.279	0.068*	0.561 (15)
C1B	0.175 (2)	0.8944 (11)	−0.1821 (12)	0.033 (3)	0.439 (15)
C2B	0.0910 (16)	0.9528 (13)	−0.1515 (19)	0.028 (3)	0.439 (15)
H2B	0.0097	0.931	−0.1548	0.034*	0.439 (15)
C3B	0.1259 (18)	1.0430 (12)	−0.116 (2)	0.031 (4)	0.439 (15)
H3B	0.0684	1.0829	−0.0953	0.037*	0.439 (15)
C4B	0.2448 (19)	1.0749 (12)	−0.1114 (16)	0.031 (3)	0.439 (15)
C5B	0.3288 (16)	1.0166 (17)	−0.142 (2)	0.028 (3)	0.439 (15)
H5B	0.4101	1.0384	−0.1387	0.033*	0.439 (15)
C6B	0.2939 (19)	0.9263 (15)	−0.177 (2)	0.031 (3)	0.439 (15)
H6B	0.3514	0.8865	−0.1982	0.037*	0.439 (15)
C7B	0.140 (2)	0.7948 (13)	−0.2213 (16)	0.042 (4)	0.439 (15)
H7B	0.2226	0.7599	−0.1945	0.05*	0.439 (15)
C8B	0.054 (3)	0.7458 (19)	−0.160 (3)	0.049 (6)	0.439 (15)
H8B1	0.0927	0.7492	−0.0633	0.073*	0.439 (15)
H8B2	−0.03	0.7752	−0.1885	0.073*	0.439 (15)
H8B3	0.0447	0.6815	−0.1871	0.073*	0.439 (15)
C9B	0.084 (3)	0.791 (2)	−0.3714 (18)	0.047 (6)	0.439 (15)
H9B1	0.1405	0.8228	−0.4086	0.07*	0.439 (15)
H9B2	0.0743	0.727	−0.4003	0.07*	0.439 (15)
H9B3	−0.0004	0.8207	−0.4018	0.07*	0.439 (15)
C10B	0.280 (3)	1.1744 (14)	−0.072 (2)	0.040 (5)	0.439 (15)
H10D	0.2606	1.2121	−0.1511	0.06*	0.439 (15)
H10E	0.3709	1.1786	−0.0195	0.06*	0.439 (15)
H10F	0.2294	1.1963	−0.0193	0.06*	0.439 (15)
C11	0.2519 (8)	0.9918 (7)	0.2698 (9)	0.0254 (18)	
C12	0.2049 (9)	0.9871 (8)	0.3746 (10)	0.033 (2)	
H12	0.1296	0.9515	0.3575	0.04*	
C13	0.2518 (10)	1.0265 (8)	0.4988 (10)	0.037 (2)	
H13	0.2066	1.0116	0.5559	0.045*	
C14	0.3564 (11)	1.0851 (9)	0.5517 (11)	0.045 (3)	
H14	0.3716	1.1058	0.639	0.054*	
C15	0.4393 (10)	1.1163 (8)	0.4942 (10)	0.038 (2)	
H15	0.5031	1.1574	0.5459	0.045*	
C16	0.4436 (9)	1.0967 (7)	0.3722 (10)	0.032 (2)	
H16	0.5106	1.1266	0.3528	0.038*	
C17	0.3659 (8)	1.0398 (7)	0.2702 (9)	0.0256 (18)	
O1	0.1888 (6)	0.9495 (5)	0.1605 (6)	0.0312 (15)	
O2	0.3948 (6)	1.0300 (5)	0.1647 (6)	0.0287 (14)	
Br1	0.40095 (10)	0.81751 (8)	0.13655 (12)	0.0417 (3)	
Os1	0.26979 (3)	0.95325 (3)	0.01352 (3)	0.02377 (12)	

### Atomic displacement parameters (Å^2^)

**Table:**

	*U*^11^	*U*^22^	*U*^33^	*U*^12^	*U*^13^	*U*^23^
C1A	0.019 (6)	0.056 (6)	0.031 (6)	0.004 (5)	0.011 (5)	0.007 (5)
C2A	0.018 (6)	0.061 (7)	0.022 (6)	−0.002 (6)	0.005 (5)	0.006 (7)
C3A	0.019 (5)	0.064 (8)	0.024 (6)	−0.007 (6)	0.007 (5)	0.001 (7)
C4A	0.028 (6)	0.060 (7)	0.015 (6)	−0.003 (5)	0.007 (5)	0.000 (6)
C5A	0.021 (5)	0.059 (6)	0.022 (7)	0.002 (5)	0.008 (6)	−0.001 (6)
C6A	0.025 (5)	0.056 (5)	0.022 (6)	0.002 (5)	0.016 (5)	0.008 (6)
C7A	0.029 (9)	0.055 (6)	0.048 (8)	0.000 (6)	0.026 (7)	0.012 (7)
C8A	0.058 (12)	0.059 (11)	0.047 (10)	−0.001 (10)	0.034 (9)	−0.001 (9)
C9A	0.052 (11)	0.056 (10)	0.046 (9)	0.012 (10)	0.028 (9)	0.011 (8)
C10A	0.038 (10)	0.065 (7)	0.032 (9)	−0.009 (7)	0.010 (8)	−0.012 (9)
C1B	0.022 (7)	0.056 (7)	0.017 (7)	−0.003 (5)	0.002 (6)	−0.001 (6)
C2B	0.022 (6)	0.051 (8)	0.017 (6)	−0.005 (6)	0.013 (5)	0.013 (7)
C3B	0.016 (6)	0.056 (8)	0.022 (7)	0.002 (6)	0.009 (6)	0.000 (7)
C4B	0.022 (7)	0.048 (6)	0.028 (7)	−0.002 (5)	0.015 (6)	0.008 (6)
C5B	0.021 (6)	0.049 (6)	0.021 (7)	0.001 (5)	0.017 (6)	0.014 (6)
C6B	0.027 (6)	0.056 (6)	0.013 (7)	0.000 (5)	0.012 (6)	0.001 (6)
C7B	0.029 (9)	0.059 (8)	0.030 (8)	−0.004 (7)	0.003 (7)	−0.006 (8)
C8B	0.055 (14)	0.050 (12)	0.044 (11)	−0.010 (10)	0.020 (11)	−0.009 (11)
C9B	0.050 (14)	0.058 (14)	0.030 (8)	0.005 (11)	0.013 (9)	−0.013 (8)
C10B	0.030 (11)	0.051 (8)	0.047 (12)	−0.002 (8)	0.023 (10)	0.000 (9)
C11	0.017 (4)	0.038 (5)	0.023 (4)	0.007 (4)	0.009 (3)	0.004 (4)
C12	0.024 (4)	0.050 (6)	0.028 (5)	0.001 (4)	0.012 (4)	0.004 (4)
C13	0.033 (5)	0.060 (7)	0.025 (5)	0.005 (5)	0.018 (4)	0.000 (4)
C14	0.046 (6)	0.057 (7)	0.025 (5)	0.018 (6)	0.002 (5)	−0.006 (5)
C15	0.037 (5)	0.043 (6)	0.028 (5)	0.003 (5)	0.005 (4)	−0.006 (4)
C16	0.028 (5)	0.038 (6)	0.029 (5)	0.001 (4)	0.010 (4)	−0.004 (4)
C17	0.020 (4)	0.033 (5)	0.025 (4)	0.004 (4)	0.009 (3)	0.000 (4)
O1	0.019 (3)	0.053 (4)	0.024 (3)	−0.005 (3)	0.010 (3)	−0.006 (3)
O2	0.020 (3)	0.046 (4)	0.026 (3)	−0.006 (3)	0.016 (3)	−0.004 (3)
Br1	0.0316 (5)	0.0392 (6)	0.0526 (7)	0.0059 (4)	0.0128 (5)	0.0038 (5)
Os1	0.01538 (16)	0.0385 (2)	0.02000 (17)	0.00078 (14)	0.00944 (12)	−0.00243 (15)

### Geometric parameters (Å, º)

**Table:**

Os1—C1A	2.090 (12)	C1B—C2B	1.39
Os1—C2A	2.125 (14)	C1B—C6B	1.39
Os1—C3A	2.158 (14)	C1B—C7B	1.528 (10)
Os1—C4A	2.157 (12)	C2B—C3B	1.39
Os1—C5A	2.123 (18)	C2B—H2B	0.95
Os1—C6A	2.089 (18)	C3B—C4B	1.39
Os1—C1B	2.187 (13)	C3B—H3B	0.95
Os1—C2B	2.164 (17)	C4B—C5B	1.39
Os1—C3B	2.167 (19)	C4B—C10B	1.526 (10)
Os1—C4B	2.192 (16)	C5B—C6B	1.39
Os1—C5B	2.21 (2)	C5B—H5B	0.95
Os1—C6B	2.21 (2)	C6B—H6B	0.95
Os1—O1	2.088 (6)	C7B—C9B	1.527 (11)
Os1—O2	2.071 (6)	C7B—C8B	1.527 (11)
Os1—Br1	2.5472 (12)	C7B—H7B	1
C1A—C2A	1.39	C8B—H8B1	0.98
C1A—C6A	1.39	C8B—H8B2	0.98
C1A—C7A	1.528 (10)	C8B—H8B3	0.98
C2A—C3A	1.39	C9B—H9B1	0.98
C2A—H2A	0.95	C9B—H9B2	0.98
C3A—C4A	1.39	C9B—H9B3	0.98
C3A—H3A	0.95	C10B—H10D	0.98
C4A—C5A	1.39	C10B—H10E	0.98
C4A—C10A	1.528 (10)	C10B—H10F	0.98
C5A—C6A	1.39	C11—O1	1.303 (11)
C5A—H5A	0.95	C11—C12	1.408 (13)
C6A—H6A	0.95	C11—C17	1.452 (13)
C7A—C9A	1.524 (10)	C12—C13	1.388 (14)
C7A—C8A	1.531 (10)	C12—H12	0.95
C7A—H7A	1	C13—C14	1.399 (17)
C8A—H8A1	0.98	C13—H13	0.95
C8A—H8A2	0.98	C14—C15	1.362 (17)
C8A—H8A3	0.98	C14—H14	0.95
C9A—H9A1	0.98	C15—C16	1.369 (14)
C9A—H9A2	0.98	C15—H15	0.95
C9A—H9A3	0.98	C16—C17	1.413 (13)
C10A—H10A	0.98	C16—H16	0.95
C10A—H10B	0.98	C17—O2	1.299 (11)
C10A—H10C	0.98		
			
C2A—C1A—C6A	120	C5B—C6B—H6B	120
C2A—C1A—C7A	121.3 (12)	C1B—C6B—H6B	120
C6A—C1A—C7A	118.7 (12)	Os1—C6B—H6B	130.1
C2A—C1A—Os1	72.1 (6)	C9B—C7B—C8B	111 (2)
C6A—C1A—Os1	70.5 (6)	C9B—C7B—C1B	107.5 (14)
C7A—C1A—Os1	129.6 (4)	C8B—C7B—C1B	117.5 (19)
C1A—C2A—C3A	120	C9B—C7B—H7B	106.7
C1A—C2A—Os1	69.4 (6)	C8B—C7B—H7B	106.7
C3A—C2A—Os1	72.4 (4)	C1B—C7B—H7B	106.7
C1A—C2A—H2A	120	C7B—C8B—H8B1	109.5
C3A—C2A—H2A	120	C7B—C8B—H8B2	109.5
Os1—C2A—H2A	130.9	H8B1—C8B—H8B2	109.5
C2A—C3A—C4A	120	C7B—C8B—H8B3	109.5
C2A—C3A—Os1	69.8 (4)	H8B1—C8B—H8B3	109.5
C4A—C3A—Os1	71.2 (5)	H8B2—C8B—H8B3	109.5
C2A—C3A—H3A	120	C7B—C9B—H9B1	109.5
C4A—C3A—H3A	120	C7B—C9B—H9B2	109.5
Os1—C3A—H3A	131.9	H9B1—C9B—H9B2	109.5
C5A—C4A—C3A	120	C7B—C9B—H9B3	109.5
C5A—C4A—C10A	120.2 (13)	H9B1—C9B—H9B3	109.5
C3A—C4A—C10A	119.8 (13)	H9B2—C9B—H9B3	109.5
C5A—C4A—Os1	69.7 (6)	C4B—C10B—H10D	109.5
C3A—C4A—Os1	71.2 (6)	C4B—C10B—H10E	109.5
C10A—C4A—Os1	131.8 (4)	H10D—C10B—H10E	109.5
C6A—C5A—C4A	120	C4B—C10B—H10F	109.5
C6A—C5A—Os1	69.4 (4)	H10D—C10B—H10F	109.5
C4A—C5A—Os1	72.4 (5)	H10E—C10B—H10F	109.5
C6A—C5A—H5A	120	O1—C11—C12	118.3 (9)
C4A—C5A—H5A	120	O1—C11—C17	115.2 (8)
Os1—C5A—H5A	130.9	C12—C11—C17	126.4 (9)
C5A—C6A—C1A	120	C13—C12—C11	129.9 (10)
C5A—C6A—Os1	72.1 (4)	C13—C12—H12	115.1
C1A—C6A—Os1	70.6 (5)	C11—C12—H12	115.1
C5A—C6A—H6A	120	C12—C13—C14	129.0 (10)
C1A—C6A—H6A	120	C12—C13—H13	115.5
Os1—C6A—H6A	129.8	C14—C13—H13	115.5
C9A—C7A—C1A	110.9 (12)	C15—C14—C13	128.3 (10)
C9A—C7A—C8A	112.4 (16)	C15—C14—H14	115.8
C1A—C7A—C8A	112.2 (15)	C13—C14—H14	115.8
C9A—C7A—H7A	107	C14—C15—C16	129.1 (11)
C1A—C7A—H7A	107	C14—C15—H15	115.5
C8A—C7A—H7A	107	C16—C15—H15	115.5
C7A—C8A—H8A1	109.5	C15—C16—C17	131.2 (10)
C7A—C8A—H8A2	109.5	C15—C16—H16	114.4
H8A1—C8A—H8A2	109.5	C17—C16—H16	114.4
C7A—C8A—H8A3	109.5	O2—C17—C16	118.7 (8)
H8A1—C8A—H8A3	109.5	O2—C17—C11	115.5 (8)
H8A2—C8A—H8A3	109.5	C16—C17—C11	125.8 (9)
C7A—C9A—H9A1	109.5	C11—O1—Os1	116.1 (6)
C7A—C9A—H9A2	109.5	C17—O2—Os1	116.7 (6)
H9A1—C9A—H9A2	109.5	O2—Os1—O1	76.3 (2)
C7A—C9A—H9A3	109.5	O2—Os1—C6A	95.9 (5)
H9A1—C9A—H9A3	109.5	O1—Os1—C6A	155.7 (6)
H9A2—C9A—H9A3	109.5	O2—Os1—C1A	95.9 (4)
C4A—C10A—H10A	109.5	O1—Os1—C1A	118.1 (5)
C4A—C10A—H10B	109.5	C6A—Os1—C1A	38.9 (3)
H10A—C10A—H10B	109.5	O2—Os1—C5A	121.7 (5)
C4A—C10A—H10C	109.5	O1—Os1—C5A	160.9 (6)
H10A—C10A—H10C	109.5	C6A—Os1—C5A	38.5 (3)
H10B—C10A—H10C	109.5	C1A—Os1—C5A	69.7 (4)
C2B—C1B—C6B	120	O2—Os1—C2A	121.5 (5)
C2B—C1B—C7B	121.6 (16)	O1—Os1—C2A	94.5 (5)
C6B—C1B—C7B	118.4 (16)	C6A—Os1—C2A	69.7 (3)
C2B—C1B—Os1	70.5 (7)	C1A—Os1—C2A	38.5 (2)
C6B—C1B—Os1	72.5 (8)	C5A—Os1—C2A	81.8 (3)
C7B—C1B—Os1	129.3 (5)	O2—Os1—C4A	158.9 (5)
C1B—C2B—C3B	120	O1—Os1—C4A	123.4 (5)
C1B—C2B—Os1	72.2 (7)	C6A—Os1—C4A	69.0 (4)
C3B—C2B—Os1	71.4 (5)	C1A—Os1—C4A	81.7 (3)
C1B—C2B—H2B	120	C5A—Os1—C4A	37.9 (2)
C3B—C2B—H2B	120	C2A—Os1—C4A	68.4 (3)
Os1—C2B—H2B	128.6	O2—Os1—C3A	158.7 (5)
C4B—C3B—C2B	120	O1—Os1—C3A	97.2 (5)
C4B—C3B—Os1	72.4 (7)	C6A—Os1—C3A	81.7 (3)
C2B—C3B—Os1	71.2 (5)	C1A—Os1—C3A	69.0 (3)
C4B—C3B—H3B	120	C5A—Os1—C3A	68.4 (3)
C2B—C3B—H3B	120	C2A—Os1—C3A	37.9 (2)
Os1—C3B—H3B	128.7	C4A—Os1—C3A	37.6 (2)
C3B—C4B—C5B	120	O2—Os1—C2B	145.8 (6)
C3B—C4B—C10B	118.4 (16)	O1—Os1—C2B	96.3 (5)
C5B—C4B—C10B	121.6 (16)	O2—Os1—C3B	109.1 (5)
C3B—C4B—Os1	70.4 (7)	O1—Os1—C3B	95.2 (6)
C5B—C4B—Os1	72.5 (8)	C2B—Os1—C3B	37.4 (3)
C10B—C4B—Os1	129.3 (5)	O2—Os1—C1B	160.6 (6)
C6B—C5B—C4B	120	O1—Os1—C1B	122.5 (6)
C6B—C5B—Os1	71.6 (5)	C2B—Os1—C1B	37.3 (2)
C4B—C5B—Os1	70.7 (6)	C3B—Os1—C1B	67.1 (4)
C6B—C5B—H5B	120	O2—Os1—C4B	87.4 (4)
C4B—C5B—H5B	120	O1—Os1—C4B	119.8 (6)
Os1—C5B—H5B	130.2	C2B—Os1—C4B	67.1 (4)
C5B—C6B—C1B	120	C3B—Os1—C4B	37.2 (3)
C5B—C6B—Os1	71.8 (5)	C1B—Os1—C4B	78.8 (4)
C1B—C6B—Os1	70.6 (6)		
			
C6A—C1A—C2A—C3A	0	C2B—C3B—C4B—C5B	0
C7A—C1A—C2A—C3A	−179.8 (3)	Os1—C3B—C4B—C5B	55.0 (5)
Os1—C1A—C2A—C3A	−53.8 (5)	C2B—C3B—C4B—C10B	−179.9 (3)
C6A—C1A—C2A—Os1	53.8 (5)	Os1—C3B—C4B—C10B	−124.9 (6)
C7A—C1A—C2A—Os1	−126.0 (5)	C2B—C3B—C4B—Os1	−55.0 (5)
C1A—C2A—C3A—C4A	0	C3B—C4B—C5B—C6B	0
Os1—C2A—C3A—C4A	−52.4 (6)	C10B—C4B—C5B—C6B	179.8 (3)
C1A—C2A—C3A—Os1	52.4 (6)	Os1—C4B—C5B—C6B	54.0 (5)
C2A—C3A—C4A—C5A	0	C3B—C4B—C5B—Os1	−54.0 (5)
Os1—C3A—C4A—C5A	−51.8 (5)	C10B—C4B—C5B—Os1	125.9 (5)
C2A—C3A—C4A—C10A	179.8 (3)	C4B—C5B—C6B—C1B	0
Os1—C3A—C4A—C10A	128.1 (5)	Os1—C5B—C6B—C1B	53.6 (7)
C2A—C3A—C4A—Os1	51.8 (5)	C4B—C5B—C6B—Os1	−53.6 (7)
C3A—C4A—C5A—C6A	0	C2B—C1B—C6B—C5B	0
C10A—C4A—C5A—C6A	−179.8 (3)	C7B—C1B—C6B—C5B	−180.0 (3)
Os1—C4A—C5A—C6A	−52.5 (4)	Os1—C1B—C6B—C5B	−54.2 (5)
C3A—C4A—C5A—Os1	52.5 (4)	C2B—C1B—C6B—Os1	54.2 (5)
C10A—C4A—C5A—Os1	−127.4 (5)	C7B—C1B—C6B—Os1	−125.8 (6)
C4A—C5A—C6A—C1A	0	C2B—C1B—C7B—C9B	93.9 (19)
Os1—C5A—C6A—C1A	−53.8 (5)	C6B—C1B—C7B—C9B	−86.1 (19)
C4A—C5A—C6A—Os1	53.8 (5)	Os1—C1B—C7B—C9B	−176.3 (18)
C2A—C1A—C6A—C5A	0	C2B—C1B—C7B—C8B	−32 (2)
C7A—C1A—C6A—C5A	179.8 (3)	C6B—C1B—C7B—C8B	148 (2)
Os1—C1A—C6A—C5A	54.5 (4)	Os1—C1B—C7B—C8B	58 (3)
C2A—C1A—C6A—Os1	−54.5 (4)	O1—C11—C12—C13	−178.9 (11)
C7A—C1A—C6A—Os1	125.3 (5)	C17—C11—C12—C13	1.2 (18)
C2A—C1A—C7A—C9A	−93.8 (15)	C11—C12—C13—C14	3 (2)
C6A—C1A—C7A—C9A	86.4 (16)	C12—C13—C14—C15	−1 (2)
Os1—C1A—C7A—C9A	174.1 (14)	C13—C14—C15—C16	−2 (2)
C2A—C1A—C7A—C8A	32.8 (16)	C14—C15—C16—C17	0 (2)
C6A—C1A—C7A—C8A	−147.0 (15)	C15—C16—C17—O2	−176.9 (11)
Os1—C1A—C7A—C8A	−59 (2)	C15—C16—C17—C11	4.9 (18)
C6B—C1B—C2B—C3B	0	O1—C11—C17—O2	−4.0 (12)
C7B—C1B—C2B—C3B	180.0 (3)	C12—C11—C17—O2	176.0 (9)
Os1—C1B—C2B—C3B	55.1 (6)	O1—C11—C17—C16	174.3 (9)
C6B—C1B—C2B—Os1	−55.1 (6)	C12—C11—C17—C16	−5.8 (16)
C7B—C1B—C2B—Os1	124.8 (6)	C12—C11—O1—Os1	−176.6 (7)
C1B—C2B—C3B—C4B	0	C17—C11—O1—Os1	3.4 (10)
Os1—C2B—C3B—C4B	55.5 (8)	C16—C17—O2—Os1	−175.7 (7)
C1B—C2B—C3B—Os1	−55.5 (8)	C11—C17—O2—Os1	2.6 (10)

### Hydrogen-bond geometry (Å, º)

**Table:**

*D*—H···*A*	*D*—H	H···*A*	*D*···*A*	*D*—H···*A*
C2*A*—H2*A*···O1^i^	0.95	2.40	3.24 (2)	148
C3*B*—H3*B*···O1^i^	0.95	2.75	3.38 (2)	124
C5*B*—H5*B*···O2^ii^	0.95	2.50	3.25 (2)	136
C6*A*—H6*A*···O2^ii^	0.95	2.71	3.39 (2)	124
C5*A*—H5*A*···O2^ii^	0.95	2.77	3.39 (2)	124

Symmetry codes: (i) −*x*, −*y*+2, −*z*; (ii) −*x*+1, −*y*+2, −*z*.
